# Thermal Aging Rheological Behavior of Magnetorheological Elastomers Based on Silicone Rubber

**DOI:** 10.3390/ijms21239007

**Published:** 2020-11-27

**Authors:** Siti Aishah Abdul Aziz, Saiful Amri Mazlan, U Ubaidillah, Norzilawati Mohamad, Seung-Bok Choi, Mohamad Amirul Che Aziz, Mohd Aidy Faizal Johari, Koji Homma

**Affiliations:** 1 Engineering Materials and Structures (eMast) iKohza, Malaysia-Japan International Institute of Technology (MJIIT), Universiti Teknologi Malaysia, Jalan Sultan Yahya Petra, Kuala Lumpur 54100, Malaysia; aishah118@gmail.com (S.A.A.A.); mohamadamirulcheaziz7@gmail.com (M.A.C.A.); mohdaidyfaizal@graduate.utm.my (M.A.F.J.); 2 International Center, Tokyo City University, 1 Chrome-28-1 Tamazutmi, Setagaya, Tokyo 158-0087, Japan; khomma@tcu.ac.jp; 3 Department of Mechanical Engineering, Faculty of Engineering, Universitas Sebelas Maret, Surakarta 57126, Indonesia; 4 National Center for Sustainable Transportation Technology (NCSTT), Bandung 40132, Indonesia; 5 Faculty of Engineering, Universiti Malaysia Sabah, Jalan UMS, Kota Kinabalu 88400, Malaysia; norzilawati@ums.edu.my; 6 Department of Mechanical Engineering, Inha University, Incheon 22212, Korea; seungbok@inha.ac.kr

**Keywords:** magnetorheological elastomer, thermal aging, phase shift angle, rheological properties, Payne effect, thermo-rheological

## Abstract

Engineering rubber composites have been widely used as main components in many fields including vehicle engineering and biomedical applications. However, when a rubber composite surface area is exposed to heat or sunlight and over a long-term accelerated exposure and lifecycle of test, the rubber becomes hard, thus influencing the mechanical and rheological behavior of the materials. Therefore, in this study, the deterioration of rheological characteristics particularly the phase shift angle (δ) of silicone rubber (SR) based magnetorheological elastomer (MRE) is investigated under the effect of thermal aging. SR-MRE with 60 wt% of CIPs is fabricated and subjected to a continuous temperature of 100 °C for 72 h. The characterization of SR-MRE before and after thermal aging related to hardness, micrograph, and rheological properties are characterized using low vacuum scanning electron microscopy (LV-SEM) and a rheometer, respectively. The results demonstrated that the morphological analysis has a rough surface and more voids occurred after the thermal aging. The hardness and the weight of the SR-MRE before and after thermal aging were slightly different. Nonetheless, the thermo-rheological results showed that the stress–strain behavior have changed the phase-shift angle (δ) of SR-MRE particularly at a high strain. Moreover, the complex mechanism of SR-MRE before and after thermal aging can be observed through the changes of the ‘in-rubber structure’ under rheological properties. Finally, the relationship between the phase-shift angle (δ) and the in-rubber structure due to thermal aging are discussed thoroughly which led to a better understanding of the thermo-rheological behavior of SR-MRE.

## 1. Introduction

Engineering rubber products are broadly used in many industrial areas because of their excellent physical properties, due to their durability, and their ability to deform strength. The engineering rubber products such as rubber composite have been widely used in aerospace [1], marine [2], and automotive applications [3,4]. It is well-known that this kind of rubber composite demonstrates a vital environmental sensitivity compared to metal structures, especially towards outdoor applications. The condition of exposure to these applications such as surrounding temperature, humidity, heat, and chemical attack will result in degradation with regard to time as well as aging issues. In general, the aging of the rubber composite is caused by chemical [5,6] and oxidative-destructive processes [7,8,9], thermal [10,11], heat, moisture, radiation [12,13], and also dynamical and mechanical strains. Among them, the two crucial factors negatively influencing the degradation or aging stability of rubber composites are temperature or heat, and the presence of an oxygen, which received from ambient condition or it is generated by their mechanical or dynamic strain test. The aging phenomenon is an irreversible process, which can significantly change the physical, mechanical and rheological properties of rubber composites. Changes of the interfacial adhesion between reinforcement and rubber thus cause rubber composite hardening, leading to the embrittlement and reduced lifespan of rubber composites [14].

Other than ordinary uses of rubber products in conveyer belts, tires, and other specialized rubber products, a fast development of rubber composites in the form of sensors, as well as magnetic and electromagnetic field shielding materials has been demonstrated in recent years. This kind of application requires a new type of rubber composite compound with non-conventional fillers integrated as active components. A possible way of preparing this new generation of materials is by the incorporation of magnetic particle as a filler [15,16,17]. Thus, the uniqueness of the new generation of materials namely magnetorheological elastomer (MRE) have been highlighted by the ability to exhibit an excellent flexibility, easy formability and capability to displace on a large scale in the presence of magnetic field. Nevertheless, despite their excellent properties, flexibility, and easy formability, MREs are still prone to a deterioration depending on their service environment, which can eventually lead to their failure. It is generally recognized that the thermo-mechanical deterioration such as aging of MREs will seriously affect their mechanical properties and durability, and cause early damage [18,19]. As such, a study by Kruzelak et al. [18] assessed the influence of thermooxidative aging on the properties of rubber magnetic composites, namely natural rubber (NR) and acrylonitrile-butadiene rubber (NBR), as matrices with different concentrations from 0 to 100 phr of strontium ferrite. The results demonstrated an enhancement of crosslink-density of these magnetic composites due to the formation of oxidation of the rubber chains during thermo-oxidative aging. Moreover, the changes of mechanical properties related to modulus, and hardness of rubber magnetic composite were also observed as their modulus increased up to 25% as compared to an unaged sample. Cvek et al. [20] investigated the degradation process of thermoplastic elastomers (TPEs) and TPE-based MRE with an operating twin-screw counter-rotating temperature of 185 °C. They found out that thermo mechanical degradation did enhance the stiffness of the TPE based MREs, but at the same time resulted in the 10% decrement of MR effect. Masbowski et al. [21] studied the thermooxidative aging on MRE samples of ethylene-octene copolymer filled with carbonyl iron particles (CIPs) and carbon black. The samples were subjected to a conventional drying oven with an air circulation at an elevated temperature of 70 °C for 14 days. The authors found that the mechanical properties of MRE were slightly deteriorated as the result of the thermo-oxidative aging process. However, the incorporation of CIPs did not accelerate the aging process, which might be due to the absence of various degrees of iron oxidation, i.e., Fe^2+^ and Fe^3+^ ions on the surface of vulcanizates, which inhibited the degradation of the elastomeric composites.

Moreover, numerous studies by other researchers [5,6,10,19] have concluded that, with the increased of temperature and aging time, the mechanical properties such as hardness, tear strength, and tensile strength would decrease as high as 72%. The obtained result was due to the changes of the molecular configuration accompanied by a hydro-thermal deterioration, which led to the development of microcracks of the matrix and the filler/matrix interface. However, a contradictory effect has been demonstrated in some studies [5,6], where the rubber composites exhibited a higher modulus of up to 215% in aged samples as compared to an unaged sample. Moreover, the maximum flexural stress was also increased by up to 15% and tended to decrease to almost 275% in comparison to the unaged sample. Thus, the aged rubber compound has influenced the mechanical properties based on the changes of chemical due to aging. Hence, this kind of material needs to be designed in consideration of the influence of a few environmental factors related to temperature to ensure the reliability and safety of rubber composite/MRE structures.

Based on the previous reports mentioned above, the most current studies for rubber composite/MRE are limited to the specific rubber matrices and mainly focused on crosslinking and mechanical properties. While no information has been provided regarding the thermo-rheological properties, especially the storage modulus and damping properties (tan δ), which are related to the phase changes of the shift angle (δ) related to stress–strain behavior during shear-mode. The changes on the phase angles is very important to indicate the differences in surface or underlying properties in terms of stiffness, viscoelasticity, and adhesion depending on the load applied and material test failure [22,23]. Moreover, it should be noted that these properties contribute to the energy dissipation (tan δ). The complexity of the mechanism of aging due to a few factors related to temperature, heat, or moisture, as well as micrograph changes through MREs need to be studied. Therefore, this paper presents the effects of aging condition related to continuous temperature on the rheological properties of SR based MRE (SR-MRE) particularly the changes of phase shift angle (δ) due to stress–strain. Thermal aging of the SR-MRE was conducted in a limited experimental range using a hot-air oven chamber. The effects of a continuous temperature condition on SR-MRE samples before and after aging conditions towards the changes of micrograph, as well as mechanical and rheological properties were systematically evaluated. Simultaneously, variations of the rubber hardness and rheological properties of the SR-MREs and so forth, according to the thermal aging conditions, were measured and analyzed as well as their micrograph changes.

## 2. Results and Discussion

### 2.1. Weight of SR-MRE

The results of two SR-MRE samples from the same sample preparation batch are shown in Table 1, showing almost the same weight changes, before and after thermal aging, which confirmed the consistency of the data. The weight loss of the SR-MRE was calculated as follows:

(1) $$\frac{Value\;after\;thermal\;aging-value\;before\;thermal\;aging}{Value\;before\;thermal\;aging}\times 100\%$$

**Figure 1:**
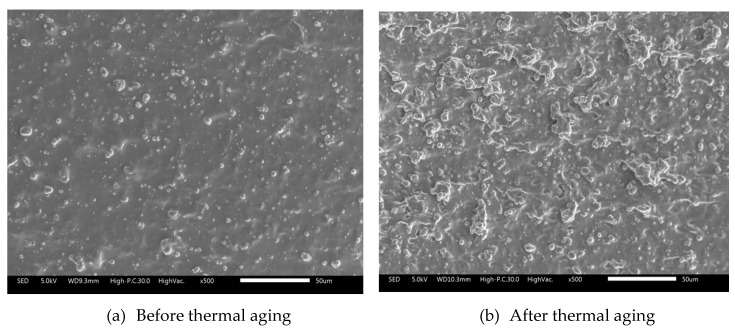
Cross-section of SR-MRE (**a**) before thermal aging and (**b**) after thermal aging.

As shown in Table 2, the samples are having weight loss of approximately 1.18% after 72 h of continuous thermal aging. The weight loss of SR-MRE after thermal aging might probably attribute to the reorientation of matrix-particles and low molecular weight owing to the continuous high temperature and degradation phenomenon occurred during thermal aging. The influence of thermal aging is well known for changing the flexibility of the polymer chains inside the SR-MRE sample, and thus resulted in more chains movement by leaving spaces between them [24]. As shown in Figure 1, cross-section surfaces of the SR-MRE gross micrograph are observed both before and after thermal aging. The SEM micrograph after the thermal aging as shown in Figure 1b is more likely roughened over the degradation period as a comparison to Figure 1a. This can be correlated with some physically changes due to the chain’s movement occurred in the SR-MRE after thermal aging. The details of the cross-section micrograph of SR-MRE are discussed in detail in next section.

### 2.2. Indentation Hardness Test of SR-MRE

The indentation hardness of SR-MRE before and after thermal aging is shown in Table 2. The hardness of SR-MRE after thermal aging has depleted by 7.25% as compared to before thermal aging. The lower number of hardness of SR-MRE sample after thermal aging was the indication of ‘soft’ materials, which might be due to the weak molecular chain owing to continuous heat exposure [25]. When a sample was exposed at an elevated temperature, the intermolecular chain experienced a coherency losing and tended to lose their interaction, and thus weakened the interaction between matrix (SR) and filler (CIPs) and resulted in the decrement of the hardness [26].

### 2.3. Micrograph Analysis

**Figure 2:**
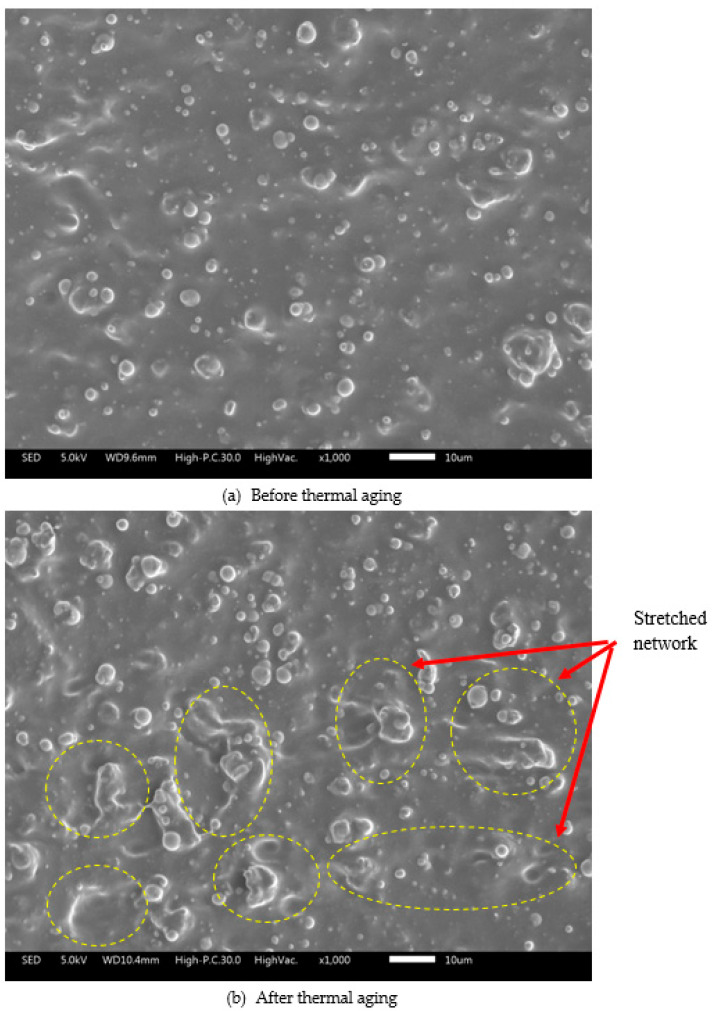
Micrograph analysis of SR-MRE (**a**) before and (**b**) after thermal aging.

The micrograph analyses of SR-MRE are shown in Figure 2a,b. The SEM images were taken at various positions across the cross-section of the samples, yet no obvious differences were observed in the SR-MRE sample either before or after thermal aging conditions. As shown in Figure 2a, a smooth cross-section of SR-MRE with uniformly dispersed and embedded CIPs in SR has been observed. The distribution of CIPs was accompanied with a small agglomeration in the cross-section area of the SR-MRE samples before thermal aging.

Even though, some small agglomerations could be observed in the SR-MRE samples, however, after the thermal aging, more agglomeration of CIPs was detected as a result of the cross-section changes in sample, which can be clearly seen in Figure 2b. Moreover, the surface of the SR-MRE after thermal aging became rougher and more spatially isolated voids were also observed. The changes of the cross-section surface and the increment of the agglomeration number of CIPs in SR-MRE might be due to the excessive cross-linking degree of SR-MRE, which resulted in a more stretched network. Moreover, this phenomenon can be related to emission of volatile organic compounds (e.g., plasticizers, thermal degradation products) from a rubber compound. This finding is almost similar to the one previously reported in work [27].

### 2.4. Rheological Properties

**Figure 3:**
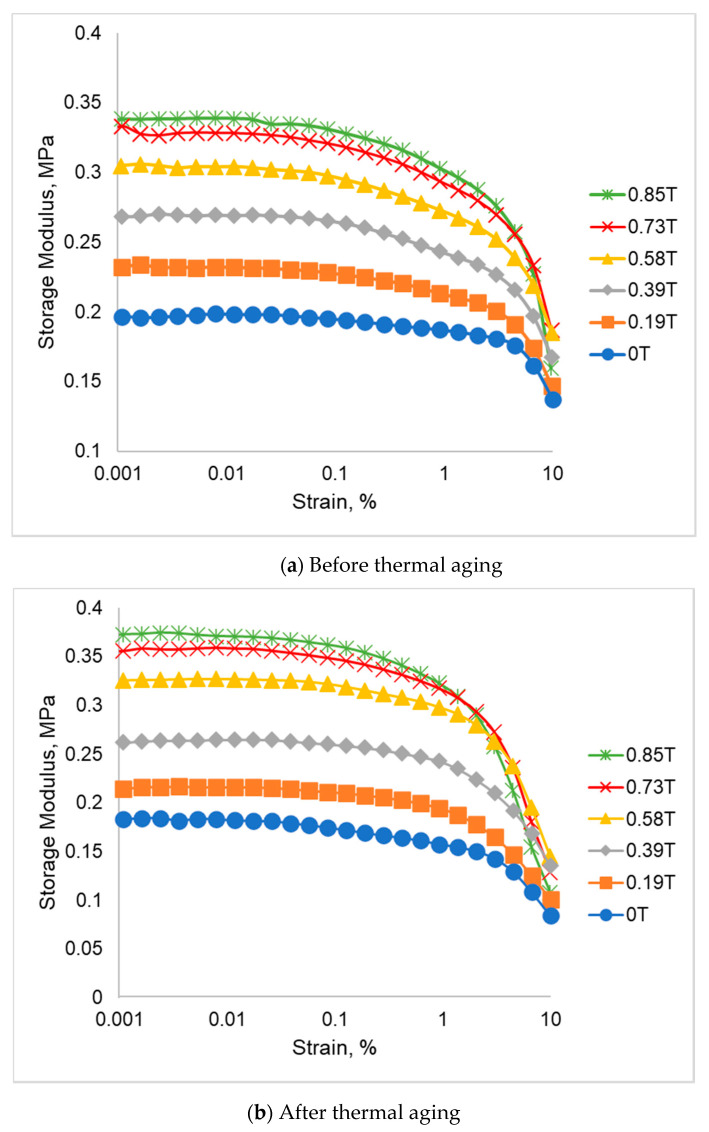
Storage modulus of SR-MRE (**a**) before and (**b**) after thermal aging.

The storage modulus of SR-MRE samples before and after thermal aging are depicted in Figure 3. As shown in Figure 3a, for the SR-MRE before thermal aging, it is clearly observed that the initial storage modulus increased parallel with an increment of magnetic field. As such, an initial storage modulus of SR-MRE at 0 T (absence of magnetic field) was 0.1997 MPa and increased with a presence of magnetic field of 0.85 T, which was up to 0.3382 MPa. In contrast, the storage modulus tended to decrease with the increment of shear strain by more than 1%. The decrement of the storage modulus was due to the Payne effect, which was particularly related to the breakdown and rearrangement of the filler (CIPs).

**Figure 4:**
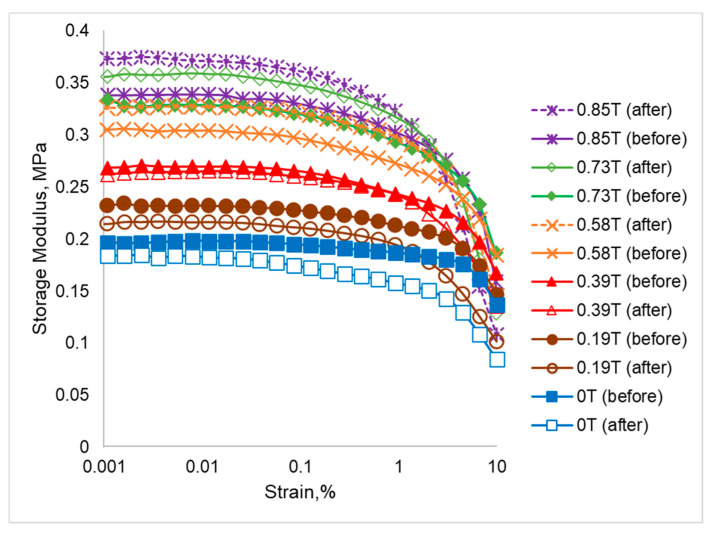
Comparison of storage modulus of SR-MRE before and after thermal aging at off- and on-state condition.

Meanwhile, Figure 3b depicts the changes of storage modulus after the thermal aging. As shown in Figure 3b, the storage modulus increases with the increment of magnetic field. The initial storage modulus in the absence of magnetic field (0 T) was 0.1838 MPa and increased up to 0.3727 MPa with the increment of magnetic field at 0.85 T. Nonetheless, the storage modulus of SR-MRE after thermal aging decreased with the increment of strain particularly at strain above 1%, which could be correlated to the Payne effects phenomenon. An interesting phenomenon was observed for both MRE before and after thermal aging whereby, at a higher magnetic field of 0.85 T, the storage modulus tends to drop at higher strain and the maximum storage modulus is lower than the magnetic field of 0.39 T. This phenomenon might be due to the high deformation and high force generated from the magnetic field that increased the particle collision between CIPs, thus disturbing the flow of the electrons and resulting in more collisions between atoms and other electrons. This condition will result in the decrement of storage modulus particularly at higher strain and higher magnetic field. The comparison of storage modulus behavior of SR-MRE before and after thermal aging are shown in Figure 4.

Figure 4 represents the comparison of storage modulus before and after thermal aging in the absence and presence of magnetic field. Interestingly, the storage modulus of SR-MRE showed a big gap, considered in a positive direction between before and after the thermal aging, where the initial storage modulus after aging was lower than before aging, for example at 0 T. As the magnetic field increased (0.19 and 0.39 T), the gap reduced till there was no gap between them. Then, by increasing more magnetic fields (0.58, 0.73 and 0.85 T), the gap was increased, but in the opposite direction, where the initial storage modulus after aging was higher than before aging.

At low magnetic fields, the interaction between CIPs was weak, and thus decreased the strength of the polymer chain due to degradation issues. This kind of phenomenon might be due to the decrement of cross-linking density that is related to the chain scission in the network at a high temperature. The formation of the chain scission might be attributed to a constitution of linear molecules in the network, which led to the slipping of matrix-matrix and particles-particles in the amorphous regions [28]. Meanwhile, a steady state occurred at one point in between 0.39 and 0.58 T of the magnetic field, where the magnetization of the magnetic particles and local field strength were at equilibrium. Later, in the presence of high magnetic fields, a significant negative trend (opposite direction) was observed as a higher storage modulus was observed in SR-MRE after thermal aging. This kind of unique trend might be due to the few phenomena’s, which are related to the matrix-matrix, filler-filler and matrix-filler interactions. It is well-known that, in this condition, the magnetic particles are subjected to a strong attractive force between neighboring particles along the field direction and a repulsive force normal to it. There are several possible explanations for this result. In the absence of a low magnetic field, the storage modulus after thermal aging was lower compared to before thermal aging, which can be correlated to the Rayleigh law, i.e., the dependence between the magnetic flux density within the material and the magnetic field. The exposure of SR-MRE towards continuous high temperature might result in some ‘defects’ inside the materials. Therefore, at this condition, the magnetic domain of the CIPs tended to be aligned in the direction of the applied field. However, due to the ‘defects’ that occurred inside the materials, the domains wall motion of CIPs was hindered and led to the decrement of storage modulus. As has been reported elsewhere, the mobility of the magnetic particles inside the polymer matrix is commonly associated with the changes of the storage modulus behavior [29,30,31]. Meanwhile, the increment of storage modulus was more pronounced at higher magnetic fields after thermal aging. The presence of free volume in the matrix–filler interfaces, as well as extended freedom movement inside the SR-MRE, might contribute to the increase in mobility of the polymer chains and magnetic particle interaction, as well as magnetic domain motion. It could therefore be assumed that the enhancement of storage modulus was related to the decrement of particle distance and stronger magnetic interaction when the magnetic field was increased.

**Figure 5:**
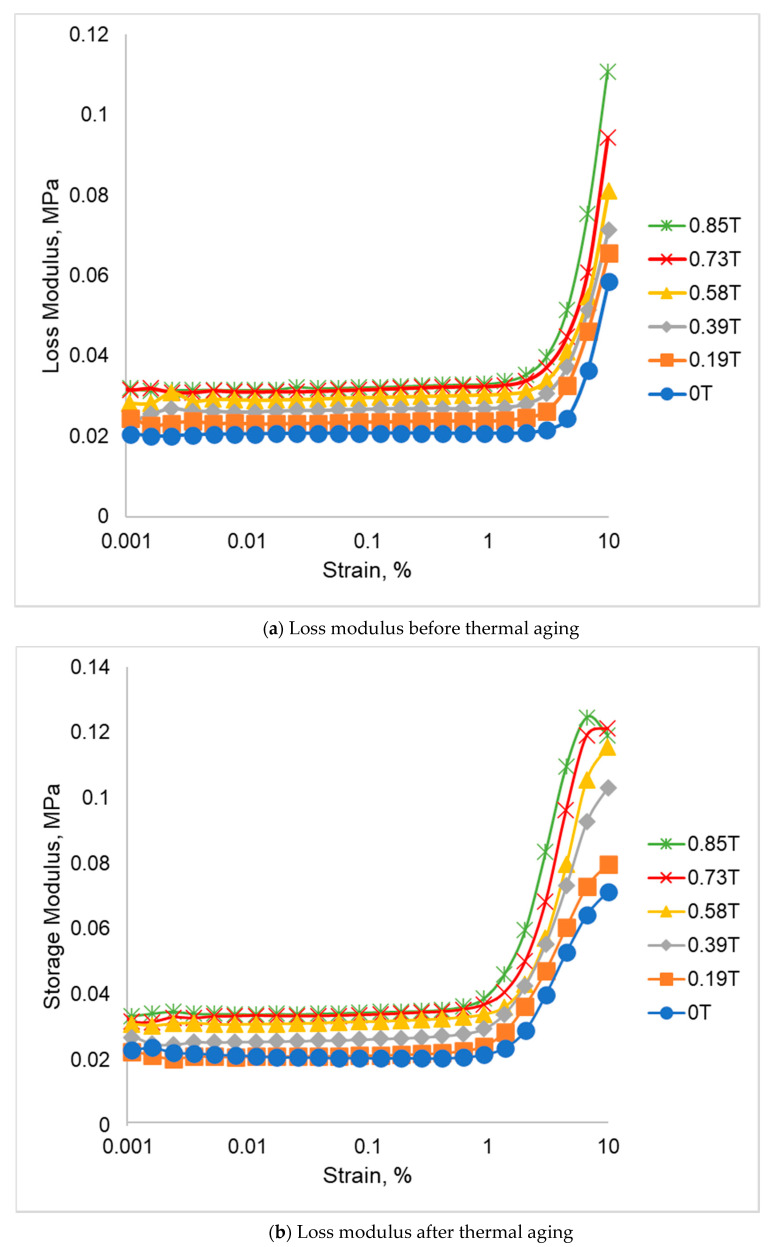
Loss modulus vs strain (**a**) before thermal aging and (**b**) after thermal aging.

The loss modulus of SR-MRE before and after thermal aging are shown in Figure 5. The loss modulus represents energy dissipated in the form of heat when the material is under stress/strain condition. As demonstrated in Figure 5a, at 0 T, the loss modulus is increased slightly with the increase of strain until about 5% strain, and then the curve further drastically increases until 10% strain. The same trend was also observed in the presence of a magnetic field as there was a gradual increase of initial loss modulus of SR-MRE with the increment of magnetic field. As such, the initial loss modulus of SR-MRE at 0T was 0.021 MPa and increased slightly up to 0.032 MPa with the increment of magnetic field up to 0.85 T. Moreover, what can be clearly seen in this figure is the continual growth of the loss modulus of SR-MRE at higher strains, i.e., greater than 5%. Likewise, the curves experienced an exponential increase of maximum loss modulus of SR-MRE at 10% of strain with the increment of magnetic field. For example, the maximum loss modulus obtained from this study at 0 T was 0.059 MPa and increased approximately 88% at a high magnetic field of 0.85 T.

Concurrently, the loss modulus of SR-MRE after thermal aging is depicted in Figure 5b. The increase of initial loss modulus was observed in all magnetic fields. Moreover, a significant change of loss modulus was also observed in all SR-MRE samples at a higher strain of more than 5%. The summary of minimum and maximum loss modulus of SR-MRE before and after aging is tabulated in Table 3.

The minimum loss modulus changes of SR-MRE samples before and after thermal aging are insignificant, starting from 0 to 0.39 T. The result suggested that there was no significant structural changes or temperature transition that affect the strain dependence of the loss modulus. However, some small changes up to 3% of minimum loss modulus were observed after the thermal aging at a magnetic field of 0.58 to 0.85 T. In contrast, the maximum of loss modulus showed a significant change after thermal aging in all magnetic fields. For example, at magnetic field of 0.39 T, a pronounced increment of maximum loss modulus up to 45% was observed after the thermal aging. The higher loss modulus at high strains greater than 5% might be associated with the SR-MRE in-structure system in which the matrix tended to have a stretched network, structural rearrangement, entanglement of filler network, or more agglomeration of CIPs that agreed well with the micrograph analysis in Figure 2b. Moreover, the changes of the in-structure system might obstruct the alignment of the matrix-matrix, matrix-particle, and particle-particle as the strain and magnetic field increased to a certain degree and then broke down or aligned with the flow under large deformation. In this situation, more heat was released within the continuous strain and applied magnetic field.

**Figure 6:**
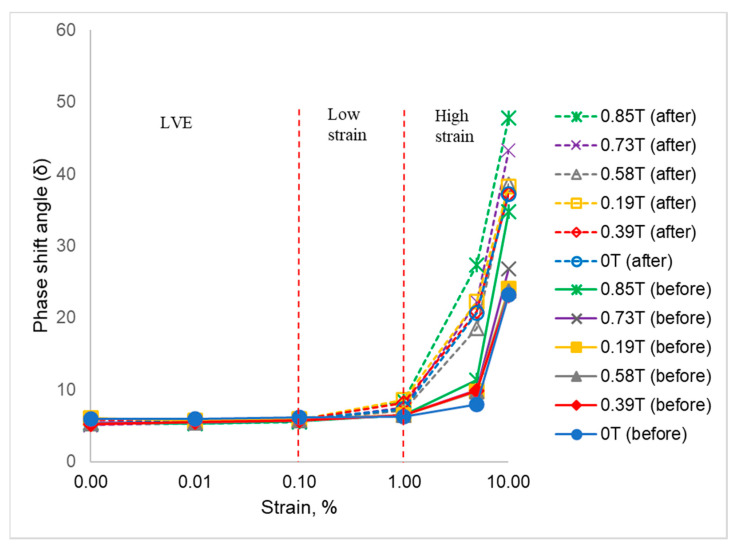
Phase shift angle (δ) at different strains and magnetic fields before and after thermal aging.

The rheological changes of the phase shift angle (δ) are one of the parameters that need to be understood because of the viscoelastic behavior. Phase lag in strain is the corresponding lag between the elastic and the viscous response in which the material deforms elastically like a solid or flows viscously like a liquid. The changes of the rheological properties between forces (stress), which generated from an external magnetic field and the changes of deformation (due to strain) can be seen from the result obtained. The comparison of the phase shift angle (δ) of all SR-MREs before and after thermal aging is shown in Figure 6.

**Figure 7:**
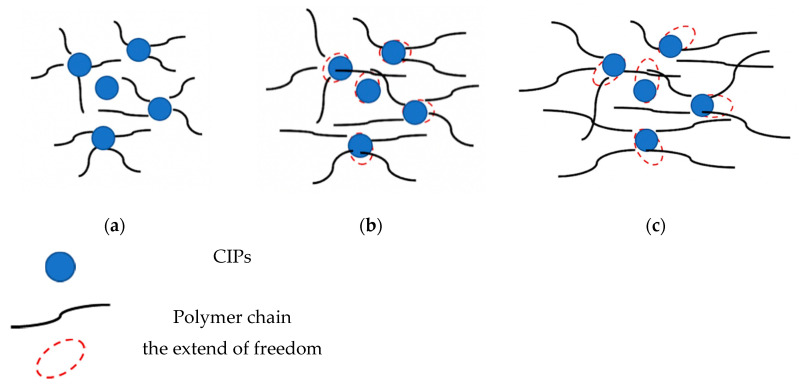
Comparison of matrix-particle interaction before thermal aging at (**a**) LVE; (**b**) low strain and (**c**) high strain regions.

Figure 6 compares the changes of phase shift angle (δ) before and after thermal aging at different strains. The phase shift angle, δ is divided into three main regions, namely LVE, low, and high strains. As for the SR-MRE before and after thermal aging, the phase shift angle (δ) depicted insignificant changes at the LVE region (0.001–0.1%). However, small changes in between before and after thermal aging were observed at a low strain region as the phase shift angle (δ) slowly increased with the increment of strain. In fact, the presence of a magnetic field also influenced the phase shift angle (δ) behavior at this region. At a high strain region (more than 1% strain), the phase shift angle (δ) remarkably increased along the strain. The increment of phase shift angle was considered small within before and after thermal aging groups at all magnetic fields. However, the difference between before and after thermal aging was obviously high. It can be clearly seen in Figure 6 that the phenomenal growth of phase shift angle (δ) after thermal aging is higher than the SR-MRE before thermal aging at all strains and magnetic fields. This increment of angle might be attributed to the changes of the chain free motion in SR as well as the force generated from the external magnetic field. Thus, an adsorption was boosted at the filler surface and influenced the matrix-particle interaction as well as the phase shift angle (δ) [1]. The plateau trend of the phase angle at LVE region both before and after thermal aging indicated the elasticity network of the samples due to strong cross-linking of SR in the presence of CIPs. With the increment of strain, the elastic response of both SR-MREs samples has been slightly modified in the absence and presence of a magnetic field. Interestingly, with a further increment of strain, the temperature influenced the elastic behavior of SR-MRE after thermal aging as the sample was more viscous. This might be associated with the occurrence of an extended freedom inside the SR matrix, depth void, and more particles agglomerations as can be clearly seen in the micrograph analysis in Figure 2. The obtained result in terms of a ‘softening’ effect due to the degradation of SR-MRE has been demonstrated by the changes of the micrograph analysis after thermal aging. This phenomenon that might attributed to the changes of matrix-filler interaction is illustrated in Figure 7.

As shown in Figure 7a, no significant changes of matrix-filler interaction are observed in the LVE region of SR-MRE before thermal aging. However, at a low strain with the presence of a magnetic field as demonstrated in Figure 7b, some changes were observed regarding the filler surface properties. A small extent of freedom occurred without altering the particle size, which agreed well with the micrograph in Figure 1b. Moreover, the changes of the surface properties can be seen as the decrease of storage modulus of SR-MRE. In this situation, the phase shift angle was slightly higher at a low strain region rather than at the LVE region. The stress–strain behavior was distinctly different due to changes of the ‘in-rubber structure’ as well as some small agglomerations. During this situation, the required stress at the same certain strain increased after torsional treatment for several times in the SR-MRE. In the meantime, the SR-MRE might also experience strain softening attributed to the void formation and breakdown of the matrix-particles network.

At a high strain, >1%, the phase shift angle was dramatically increased even before the thermal aging. The increment of the strain has changed the phase of SR-MRE as the rubber became highly stretched, which thus resulted in augmentation and a bigger void in rubber phase as demonstrated in Figure 7c. The bigger void obtained in the rubber phase obstructed CIPs movement, and simultaneously led to strain-independence that contributed to a lower modulus. When the strain was further increased, the energy dissipation was higher due to the motion of the molecular chain and larger deformation of SR. Moreover, the rubber in the materials might reach the limit of deformation and lost energy when the molecule was highly stretched.

**Figure 8:**
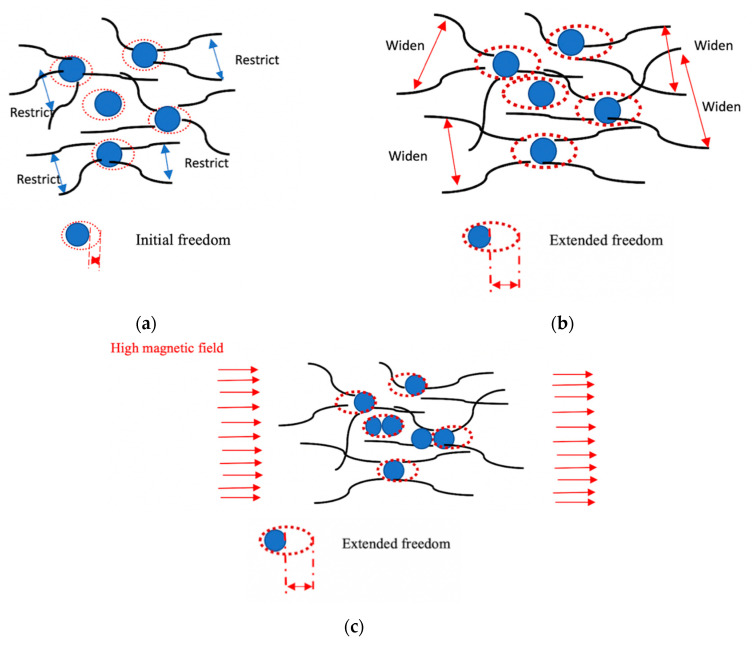
Comparison of (**a**) initial freedom; (**b**) extended freedom and (**c**) extended of freedom according to magnetic field of SR-MRE.

Nonetheless, the comparison of CIPs movement in the SR-MRE at an “initial freedom” before thermal aging and “extended freedom” after thermal aging, as well as in the presence of a magnetic field, are shown in Figure 8. As shown in Figure 8a, the initial freedom of CIPs inside the SR-MRE is very small. During this condition, the CIPs tended to have a small vibration during the shear mode condition and the stress–strain behavior was capable to maintain the elasticity condition. However, the appearance of a void caused a slight decrease in the storage modulus of the SR-MRE due to the obstructed SR and CIPs movement, which has been proven with micrograph analysis in Figure 2a. However, after the thermal aging, the SR-MRE exhibited a deeper and larger void with an extent of freedom, which can be clearly seen in Figure 8b. The figure illustrates that the rubber chain has a tendency to exhibit a bigger stretch with the increment of strain, which thus resulted in the appearance of a deep void and micro-movement of the CIPs inside the SR-MRE. It is interesting to note that, with the presence of the magnetic field after the thermal aging, the CIPs tends to vibrate, thus resulting in more attractive forces between CIPs, which can be seen in Figure 8c. An obvious strain-softening at a small deformation called the Payne effect was observed in the presence of a magnetic field. Moreover, the breakdown of the polymer chains, as well as matrix-particle and particle-particle interaction can be explained in Figure 2b as the chains tend to produce bigger stretches in this strain region. Thus, this resulted in the appearance of a deep void and micro-movement of the CIPs inside the SR-MRE. Furthermore, the size and depth appearance of the void inside the SR-MRE surface has allowed the CIPs to vibrate inside the restricted void only and ceased the deformation by allowing some agglomerations of CIPs. The result agreed well with the micrograph observation in Figure 2b. At this stage, the storage modulus SR-MRE was totally dependent on the strain.

**Figure 9:**
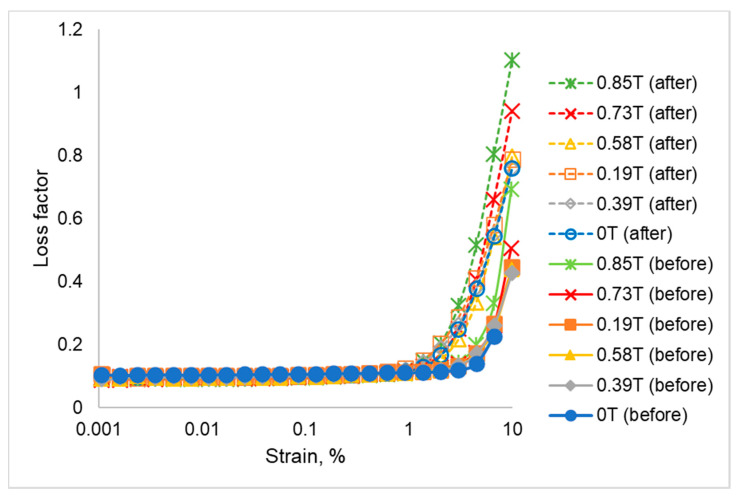
Comparison of loss factor of SR-MRE before and after thermal aging.

The loss factor (tan δ) of SR-MRE before and after thermal aging in the absence and presence of a magnetic field are demonstrated in Figure 9. The loss factor of SR-MRE before thermal aging was maintained at the same value in the LVE region. The same trend has also been observed in SR-MRE before thermal aging in the absence and presence of a magnetic field. However, no significant changes of the loss factor of SR-MRE before thermal aging were identified in a low strain region as the loss factor remained almost unchanged under the influence of a magnetic field. With a greater increment of strain as well as magnetic field, the loss factor has sharply increased.

The increment of the loss factor particularly at a high strain and in the presence of a high magnetic field was attributed to the strain hardening of the matrix chain and stronger interaction of magnetic particles due to strong attractive forces. In the absence of a magnetic field, the loss factor was weak, depending on the CIPs chains. Meanwhile, in the presence of a magnetic field, the loss factor was dominantly affected by the magnetism induced by CIPs chains. Therefore, from the obtained results, it was clearly shown that the higher the loss factor, the higher the dissipated energy due to the intermolecular friction and molecular chain relaxation. The loosening of the matrix chain, the depth void, and close attraction between magnetic particles led to an increment in the loss factor.

## 3. Methodology

### 3.1. Samples Preparation

**Figure 10:**
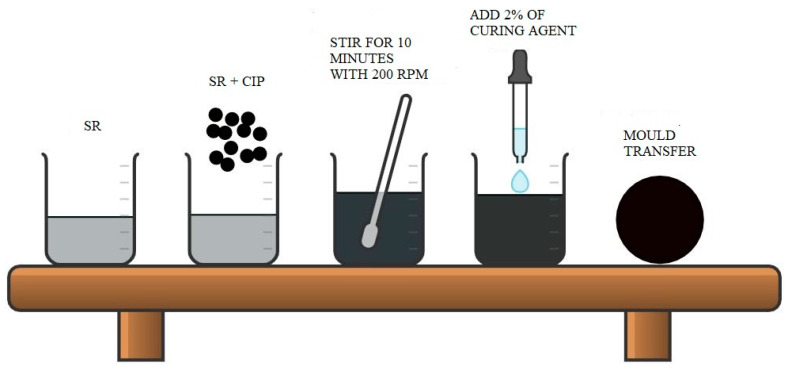
SR-MRE sample preparation.

In this study, room temperature vulcanization (RTV) silicone rubber (SR) NS 625 A, Nippon Steel from Tokyo, Japan based-MRE (SR-MRE) that consisted of 60 wt% CIPs and SR as a suspending medium was prepared. The CIPs used were a type of soft spherical magnetic particles (OM grade, BASF, Ludwigshafen, Germany) having an average size of 5 µm with a density of 7.874 g/cm^3^. The preparation sample of SR-MRE is shown in Figure 10.

The SR-MRE samples were prepared using a conventional mixing method. First, the CIPs of 60 wt% were added into the SR medium and mixed using a mechanical stirrer Multimix High Speed Dispersed (HSD). The amount of the CIPs and SR medium used in this study was based on the weight ratio. Both materials were mixed for 10 min under 200 rotation per minute (rpm) in room temperature (25 °C) until it was visually homogenous. Next, the sample was added with a curing agent, NS 625 B (Nippon Steel) with a percentage of 2% of the elastomer matrix weight and stirred for 1 min before transferring the mixture into the mold. Then, it was left for 2 h to complete the curing process. The MRE samples having a circular shape with a diameter of 20 mm and 1 mm of thickness were used as a sample in this work.

### 3.2. Sample Characterizations

The thermal aging test of SR-MRE samples were investigated using an oven chamber, Carbolite Chamber Furnace, CW 1100. The SR-MRE samples were aged in oven continuously for 72 h at a temperature of 100 °C. The aged specimens were then left for 24 h at room temperature before conducting the micrograph analysis and mechanical and rheological tests.

The indentation hardness of SR-MRE samples was determined according to ASTM D2240 using durometer (Shore-A) hardness tester. According to Brown and Soulagnet [32], hardness is essentially a measure of modulus. The SR-MRE samples were placed on a flat, smooth, and clean surface during the test. The pressure foot of the instrument was pressed onto the SR-MRE samples, making sure that it was parallel to the surface of the specimen. The durometer hardness was recorded for every 1 sec immediately after the pressure foot was in firm contact with the MRE. The determination of hardness was conducted at different positions on the sample and the average of the data was used.

The micrograph analysis of SR-MRE samples was conducted by using low vacuum scanning electron microscopy (LV-SEM), JEOL JSM-IT300 at a voltage of 5.0 kV with 2000× magnification. The cross-section of SR-MRE samples was coated with a platinum layer before performing micrograph observation in order to avoid sample charging. Meanwhile, the effect of various magnetic fields towards the rheological properties of SR-MRE samples were evaluated using a rheometer (MCR 302, Anton Paar, Graz, Austria) under an oscillation mode at room temperature. Different magnetic fields, ranging from 0 to 0.85 T, were achieved by altering the applied current from 0 to 5 A. The tests were repeated three times for the consistency of the result. In the meantime, for the sweep strain test, the strain was varied from 0.001% to 10%, with a constant frequency of 1 Hz.

## 4. Conclusions

The deterioration of rheological characteristics, particularly in the phase shift angle (δ) of SR-MRE, due to thermal aging was investigated. The study started with an experimental evaluation of the micrograph and mechanical properties of SR-MRE with 60 wt% of CIPs before and after thermal aging. The micrograph analysis revealed that there was a change in rubber structures with an appearance of a rough surface and the occurrence of voids. Meanwhile, the mechanical properties related to the hardness of the SR-MRE showed that SR-MRE after thermal aging exhibited a decrement of the hardness up to 7%. This was due to ‘soft’ SR-MRE, which was caused by the loosened interaction of the matrix-matrix and matrix-particle during the heat treatment. In the meantime, the rheological properties indicated an increase in the storage modulus of SR-MRE after thermal aging as compared to before thermal aging. The Payne effect was observed at both SR-MRE samples at all strains and with the increment of magnetic field, the storage modulus decreased due to the breakdown of the matrix chain and matrix-particle interaction. It is somewhat surprising that the temperature has influenced and changed the phase shift angle (δ), particularly at a high strain region. A rapid increment of phase shift angle was observed by the appearance of a depth void, large deformation, and particle aggregation, which are related to the matrix chain, matrix-particle, and particle-particle interaction. The finding is very interesting as the phase shift angle (δ) due to stress–strain behavior at shear-mode condition significantly increase with the temperature, thus affecting the deterioration process of SR-MRE.

## Figures and Tables

**Table 1:** Sample of SR-MRE before and after thermal aging of 100 °C for 72 h.

Sample	Before Thermal Aging (g)	After Thermal Aging (g)	Weight Loss Rate
SR-MRE (1)	1.0897	1.0767	1.19%
SR-MRE (2)	1.0911	1.0784	1.16%

**Table 2:** An average of hardness of SR-MRE before and after thermal aging by using Durometer (shore-A) hardness tester.

Sample	Before Thermal Aging		After Thermal Aging		Hardness Depletion
	Mean	SD	Mean	SD	
SR-MRE (1)	59.7	1.6	55.4	1.8	7.2%
SR-MRE (2)	57.6	3.1	53.4	2.9	7.3%

**Table 3:** Summary of loss modulus before and after thermal aging at different magnetic field.

Magnetic Field	Minimum Loss Modulus (MPa)		Maximum Loss Modulus (MPa)	
	Before Aging	After Aging	Before Aging	After Aging
0T	0.021	0.023	0.059	0.071
0.19T	0.023	0.023	0.066	0.080
0.39T	0.026	0.026	0.071	0.103
0.58T	0.028	0.031	0.081	0.116
0.73T	0.031	0.032	0.094	0.121
0.85T	0.032	0.033	0.111	0.119
